# Recurrent biliary dissemination of colon cancer liver metastasis: a case report

**DOI:** 10.1186/s13256-018-1858-x

**Published:** 2018-10-27

**Authors:** Ichiro Onishi, Masato Kayahara, Ryohei Takei, Naoki Makita, Masayoshi Munemoto, Yasumichi Yagi, Atsuhiro Kawashima

**Affiliations:** 1 Department of Surgery, National Hospital Organization Kanazawa Medical Center, 1-1 Shimoishibiki, Kanazawa, 920-8650 Japan; 2 Department of Medical Laboratory, National Hospital Organization Kanazawa Medical Center, 1-1 Shimoishibiki, Kanazawa, 920-8650 Japan

**Keywords:** Biliary dissemination, Colon cancer, Liver metastasis, Intrabiliary growth

## Abstract

**Background:**

Most colorectal cancer liver metastases form nodules within the hepatic parenchyma, and hepatectomy is the only radical treatment for synchronous metastases. There is concern about intrabiliary tumor growth which may affect the surgical margin, resulting in local recurrence after hepatectomy for colorectal cancer liver metastasis; however, there has been no report of the dissemination in the bile duct after hepatectomy. Here, we report an unusual case of biliary dissemination of colorectal cancer that caused recurrent intrabiliary growth after hepatectomy, and discuss the management of intrabiliary metastasis of colorectal cancer.

**Case presentation:**

A 69-year-old Japanese man underwent treatment for liver dysfunctions 3 years after aortic valve replacement. Computed tomography revealed an enhanced tumor within the hilar bile duct and dilatation of the left hepatic duct, typical of hilar cholangiocarcinoma. Endoscopic retrograde cholangiopancreatography revealed tumor shadow in his bile duct, and the cytology confirmed malignant cells in the bile. We performed extended left hepatectomy with bile duct resection; his postoperative course remained good without acute complications. After 3 months postoperatively, he was readmitted for subacute cholangitis and obstructive jaundice. Immediately, percutaneous transhepatic cholangiography drainage was performed, followed by cholangiography that exhibited intrabiliary tumor growth in the remnant liver. On immunohistochemical examination, tumor cells were positive for cytokeratin 20 and CDX2 but negative for cytokeratin 7. Then, computed tomography revealed an enhanced tumor-like lesion at the descending colon. After 3 months, left hemicolectomy was performed. Meanwhile, the percutaneous transhepatic cholangiography drainage fluid turned bloody, which was considered to be bleeding from a residual bile duct tumor. Accordingly, radiotherapy was initiated to prevent tumor bleeding around the hilar bile duct, but, unfortunately, the effects were short-lived, and cholangitis rebooted after 1 month leading to our patient’s death due to septic liver failure. Autopsy revealed a remnant tumor in the bile duct, but no noticeable nodular metastasis was observed, except for a single small metastasis in the lower lobe of the left lung.

**Conclusions:**

The intrabiliary growth of metastatic colorectal cancer mimics cholangiocarcinoma occasionally. To date, as the effect of chemotherapy or radiotherapy remains uncertain, the complete resection of a bile duct tumor is the only method which could result in a better prognosis.

## Background

Most colorectal cancer (CRC) liver metastases form nodules within the hepatic parenchyma, and hepatectomy is the only radical treatment for synchronous metastases [1]. Thus, a suitable cutting line is crucial during surgical resection to confirm the tumor clearance. When detected around the nodule, either macroscopically or microscopically [2, 3], intrabiliary tumor growth can affect the surgical margin, resulting in local recurrence after hepatectomy [4, 5]. However, to date, only a few cases have been reported primarily comprising development in the bile duct [5–11]; of these, many are case reports, and some are a limited number of case series. Moreover, there has been no report of recurrence in the bile duct inoculation after hepatectomy; the actual mechanism of biliary dissemination of CRC is still unknown.

Here we report a rare case of biliary dissemination of CRC which caused recurrent intrabiliary growth after hepatectomy and discuss the management of intrabiliary metastasis of CRC.

## Case presentation

A 69-year-old Japanese man underwent treatment for liver dysfunction 3 years after aortic valve replacement. Later, rapid elevation in his serum alkaline phosphatase (ALP) level was recorded and he was readmitted to determine the etiology. His body temperature was 36 °C, blood pressure 164/65 mmHg, and pulse rate was 66/minute. Laboratory data revealed mild anemia and liver-renal injury: white blood cells (WBC) 4600/uL, hemoglobin 9.7 g/dL, platelet 18.9 × 10^4^/dL, C-reactive protein (CRP) 0.29 mg/dL, ALP 1138 U/L, aspartate aminotransferase (AST) 40 U/L, alanine aminotransferase (ALT) 37 U/L, and γ glutamyl transpeptidase (γ-GTP) 298 U/L. His blood urea nitrogen (BUN) was 22.4 mg/dL, creatinine 1.14 mg/dL, activated partial thromboplastin time (APTT) 45.6 seconds, and prothrombin time-international normalized ratio (PT-INR) 2.67. He also had a past history of duodenal ulcer perforation and was currently being treated with warfarin, angiotensin receptor blocker, and proton pump inhibitor. In addition, he was taking orally administered ursodeoxycholic acid for unknown liver function disorder. He had no alcohol consumption or tobacco smoking history and no relevant family history.

**Fig. 1:**
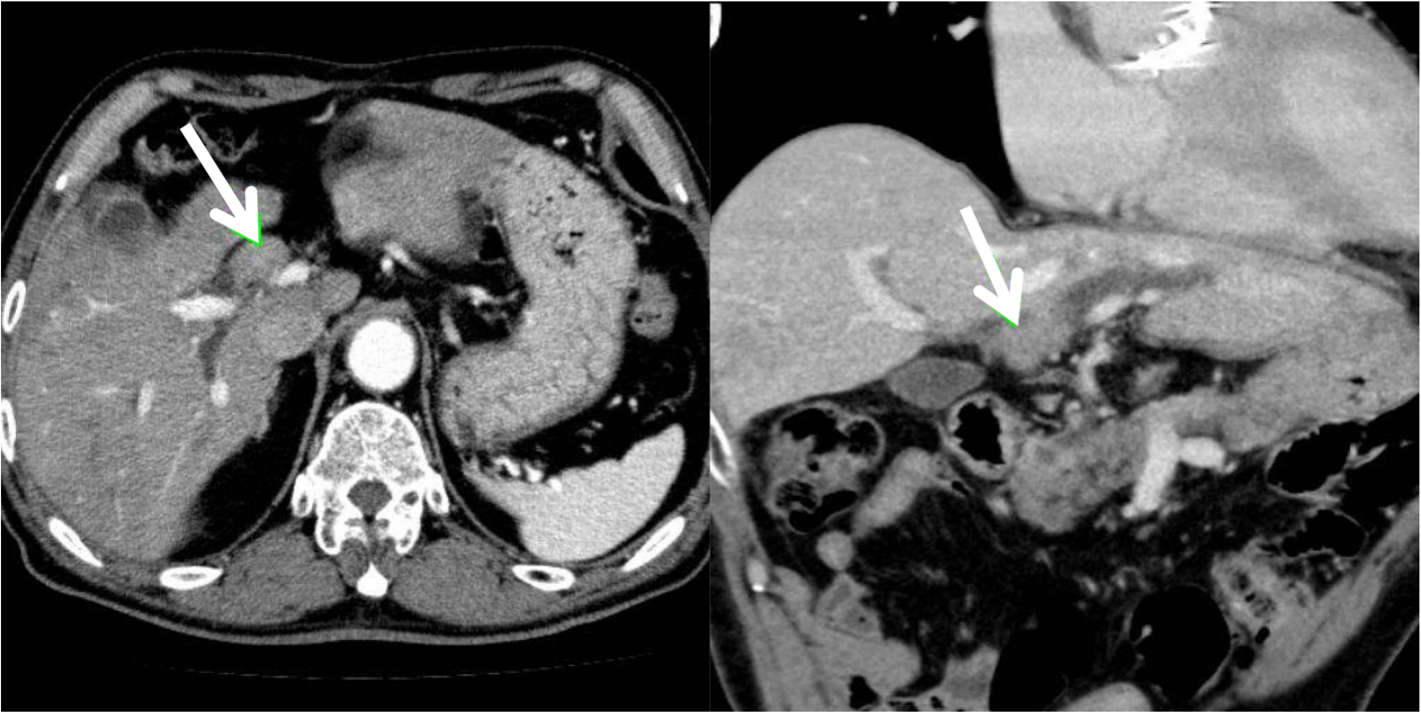
Preoperative enhanced computed tomography. Computed tomography reveals a hilar tumor (*white arrow*) and dilatation of the peripheral bile duct. No space-occupying lesion was detected in the liver parenchyma

**Fig. 2:**
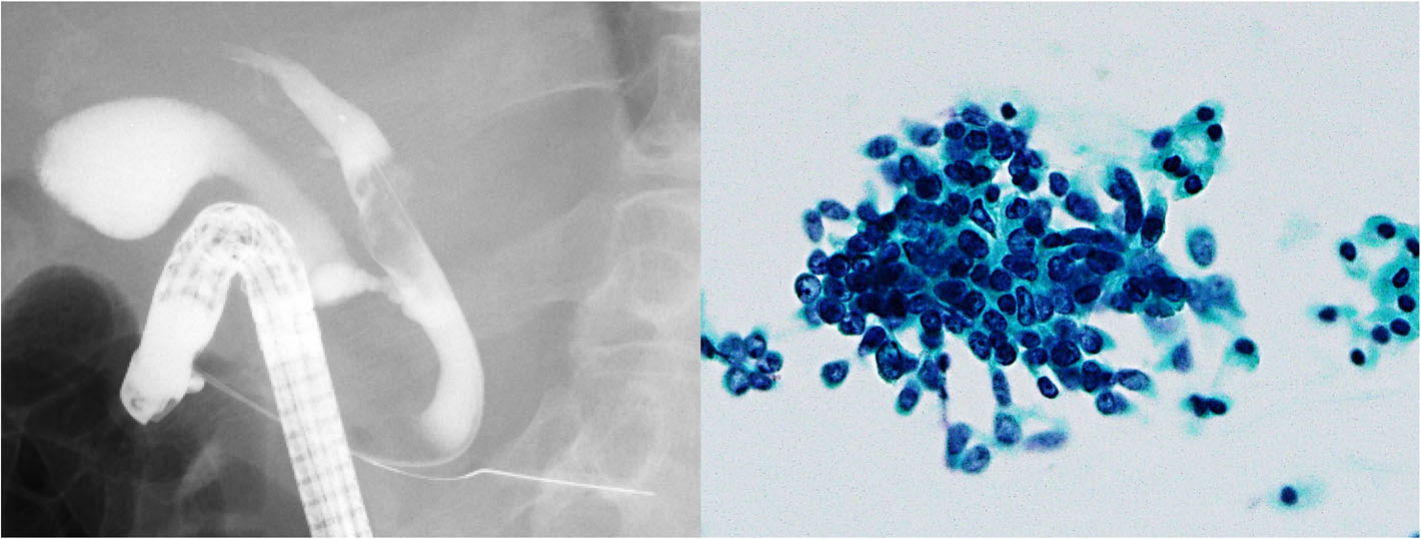
Endoscopic retrograde cholangiopancreatography and bile cytology. Endoscopic retrograde cholangiopancreatography shows the left hepatic duct obstruction and tumor shadow in the common hepatic duct. Malignant cells were observed in the bile duct cytology

A plain radiograph showed no significant findings, but computed tomography (CT) revealed an enhanced tumor within the hilar bile duct and dilatation of the left hepatic duct (Fig. 1), which are typical findings for hilar cholangiocarcinoma. In addition, endoscopic retrograde cholangiopancreatography (ERCP) revealed tumor shadow in his bile duct, and the cytology confirmed malignant cells in the bile (Fig. 2). As no lymph node and distant metastases were detected, we inserted endoscopic nasobiliary drainage (ENBD) to reduce jaundice as preparation for surgery. We performed extended left hepatectomy with resection of his bile duct; his postoperative course was good without severe complications. After 3 months postoperatively, he was readmitted for subacute cholangitis and obstructive jaundice. Immediately, percutaneous transhepatic cholangiography drainage (PTCD) was performed, followed by cholangiography that exhibited the intrabiliary tumor growth in the remnant liver.

**Fig. 3:**
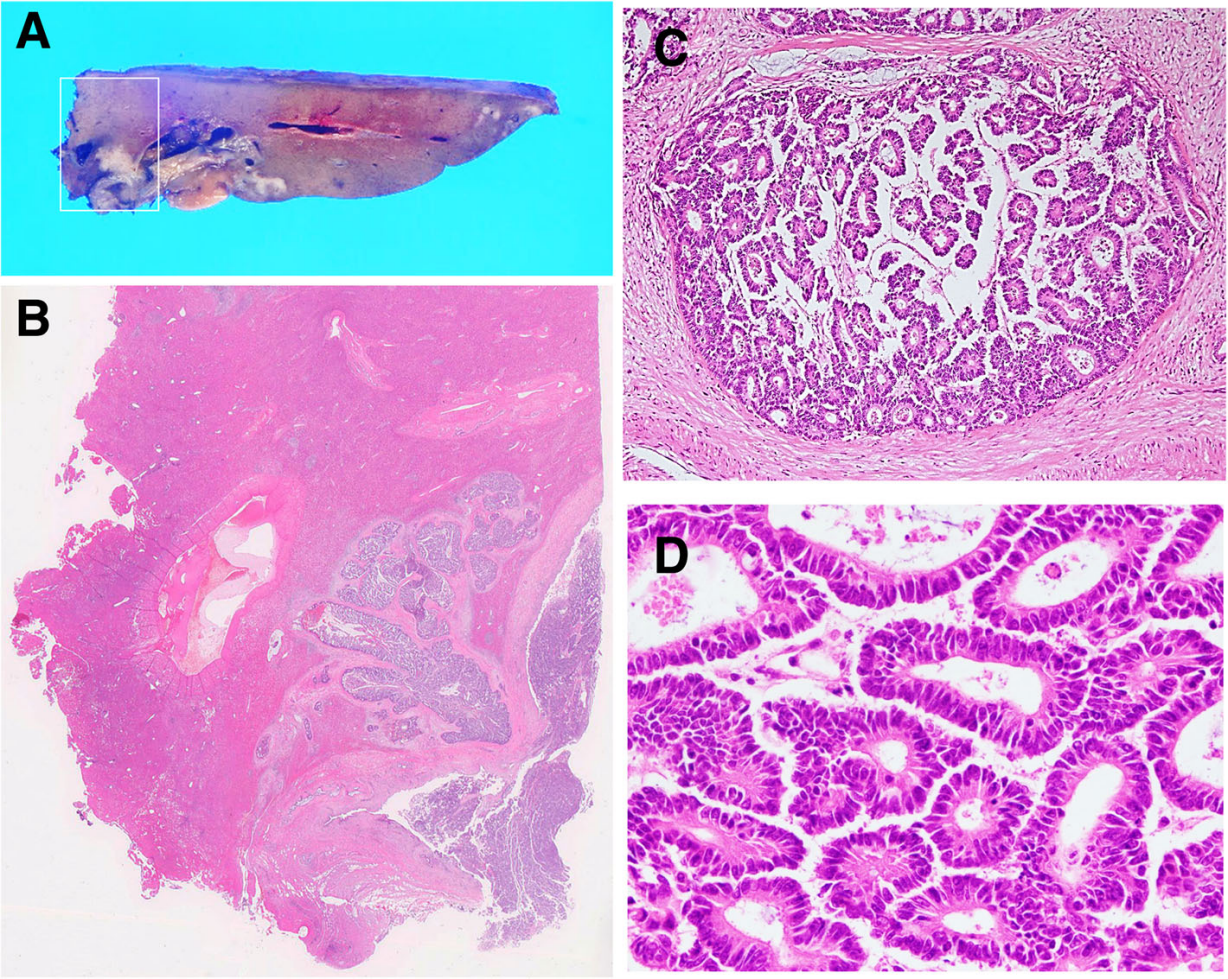
Tumor growing in the hilar duct across the left hepatic duct. The dilated bile duct was filled with well-differentiated tubular adenocarcinoma. (**a**)Macroscopic finding of resected specimens. (**b**)Loupe finding at the same site (**c**)Tumor cells along the lumen of the biliary ducts Hematoxylin and eosin, × 40 (**d**) High, magnification Hematoxylin and eosin, × 100

**Fig. 4:**
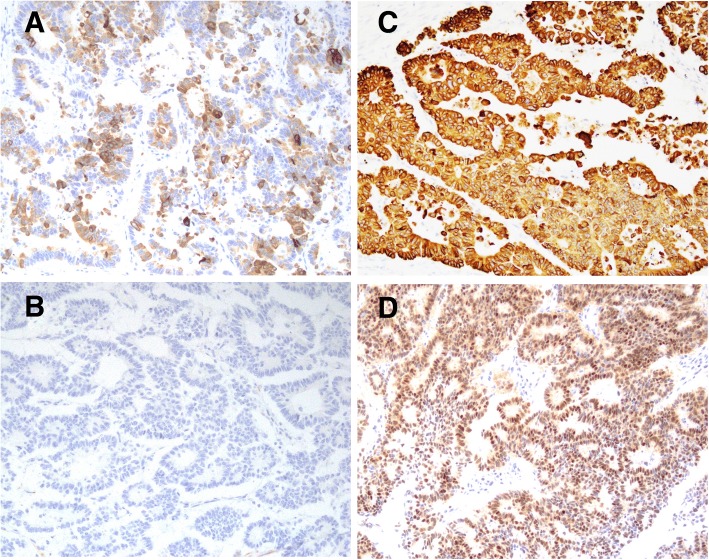
On immunohistochemical examination, tumor cells are positive for cytokeratin 20 (**a**) but negative for cytokeratin 7 (**b**). Cytokeratin 18 (**c**) as control and CDX2 (**d**) were also stained; × 100

**Fig. 5:**
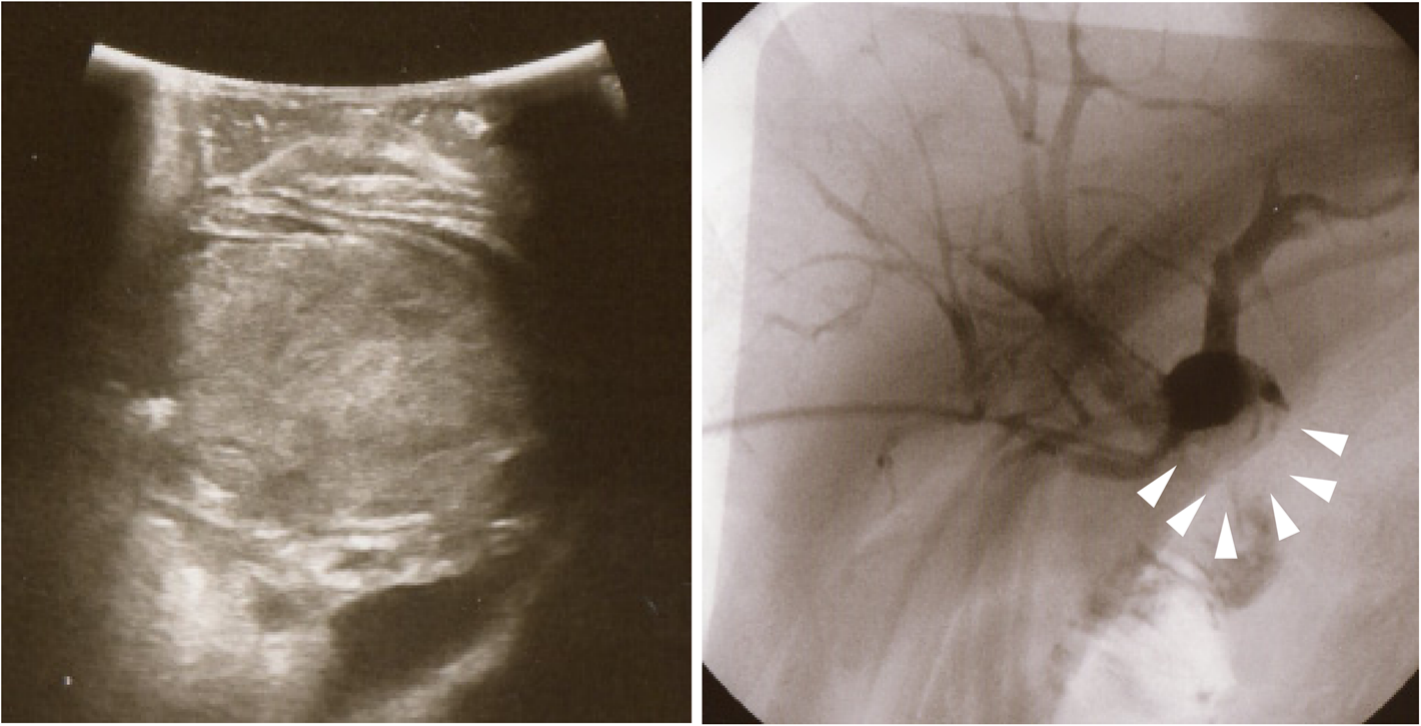
Ultrasonography showed intrabiliary growth (*left panel*) and percutaneous transhepatic cholangiography showed filling defect in the choledocojejunostomy (*arrows in right panel*)

**Fig. 6:**
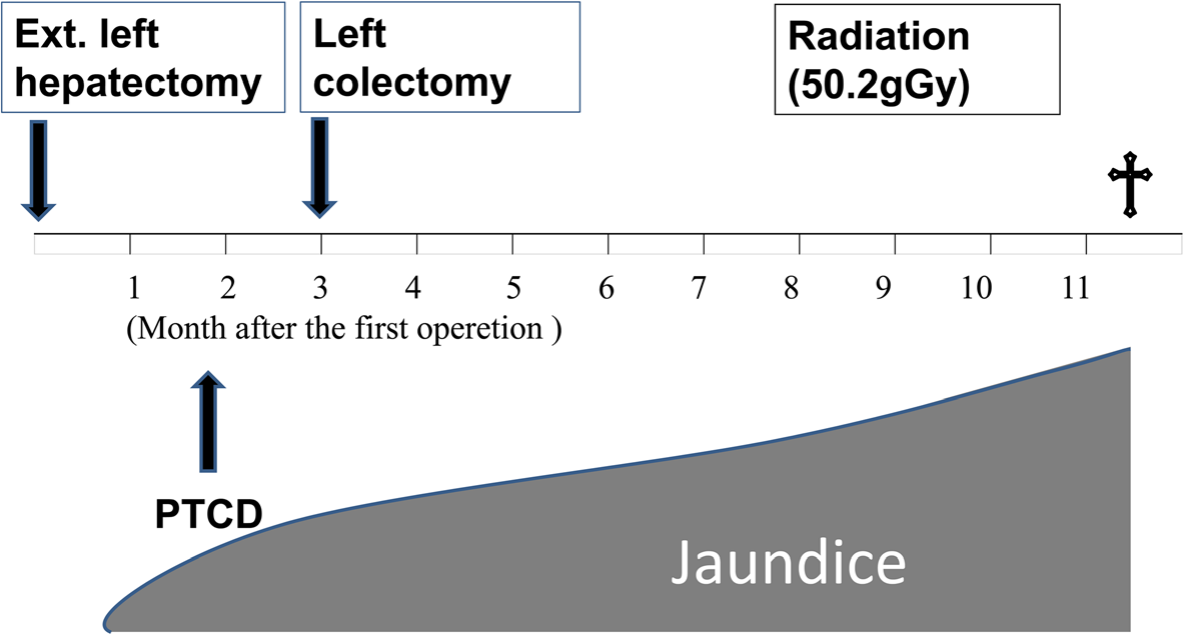
After 3 months postoperatively, he was readmitted for subacute cholangitis and obstructive jaundice. Immediately, percutaneous transhepatic cholangiography drainage was performed, followed by cholangiography that exhibited intrabiliary tumor growth in the remnant liver. Radiotherapy was initiated, but the effects were short-lived. *PTCD* percutaneous transhepatic cholangio-drainage

**Fig. 7:**
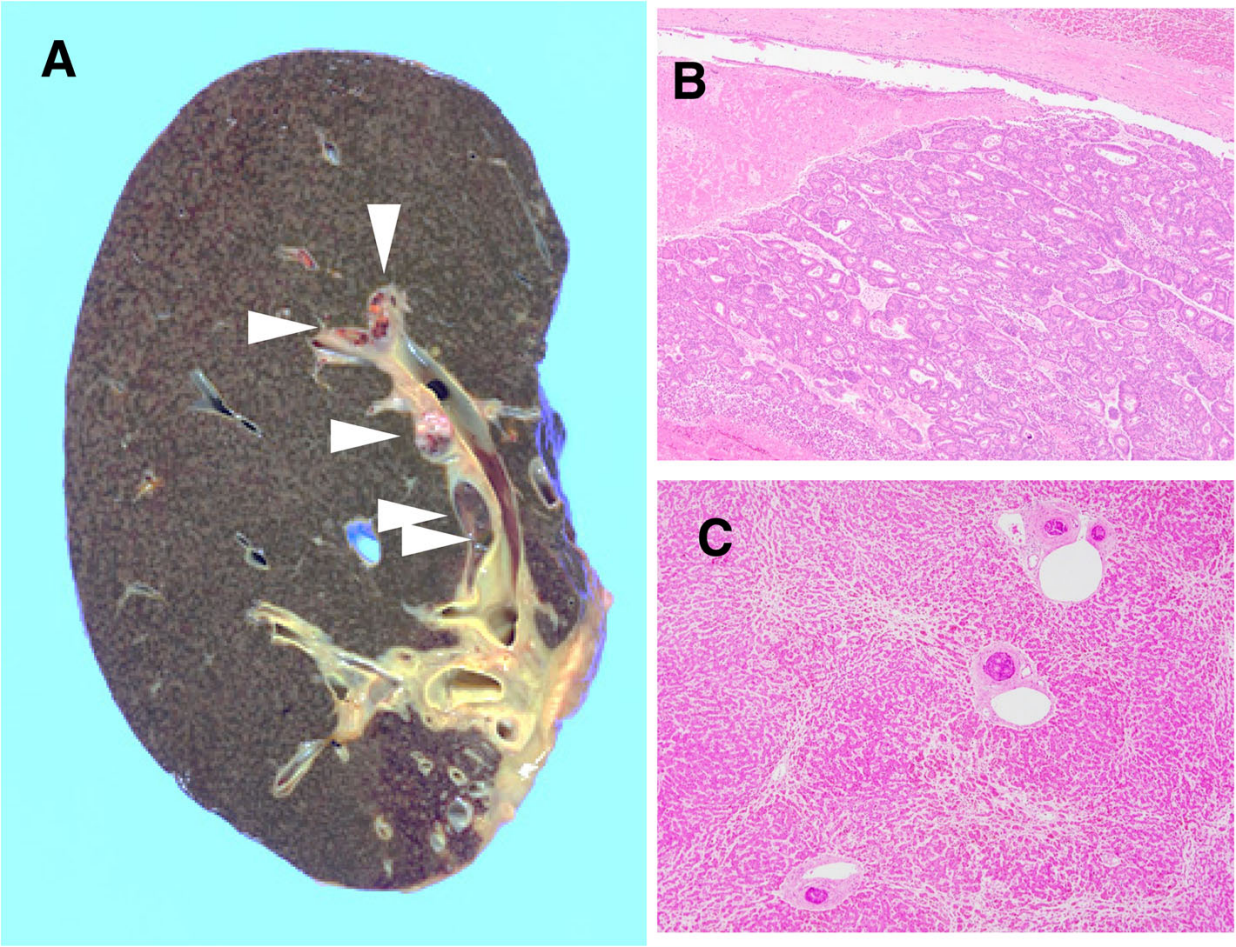
Autopsy findings of the remnant liver. Extensive cholestasis and intarabiliary tumor (*arrows*) are observed in the specimen (**a**). A well-differentiated adenocarcinoma that formed an intrabiliary growth along the lumen (**b** and **c**). Hematoxylin and eosin, × 40

Simultaneously, histological examination of resected specimens revealed tumor growth in the hilar duct across the left hepatic duct (Fig. 3a, b). Microscopic findings at the same site revealed a dilated bile duct filled with well-differentiated tubular adenocarcinoma (Fig. 3c, d). On immunohistochemical examination, tumor cells were positive for cytokeratin (CK) 20 (Fig. 4a) but negative for CK7 (Fig. 4b). Furthermore, CK18 (Fig. 4c) as control and CDX2 (Fig. 4d) were stained. Although these findings were not typical of intrahepatic cholangiocarcinoma, hepatic metastasis from another primary lesion was strongly suspected [12, 13]. Furthermore, CT revealed an enhanced tumor-like lesion at the descending colon, followed by diagnosis of type 2 cancer in total colonography. Then, left hemicolectomy was performed; the immunohistochemical-identified feature matched with an intrabiliary tumor. Meanwhile, the PTCD fluid turned bloody, which was considered to indicate bleeding from a residual bile duct tumor (Fig. 5). Accordingly, we planned chemotherapy with orally administered capecitabine but our patient experienced a spike fever because of refractory cholangitis. Thus, we abandoned chemotherapy and initiated radiotherapy to stop the tumor bleeding around the hilar bile duct. After completing radiotherapy (total 50 Gy) for approximately 1 month, we observed an improvement in his liver function because of tumor shrinkage. Unfortunately, the effects were short-lived, intrabiliary growth and cholangitis rebooted after 1 month leading to his death due to septic liver failure (Fig. 6). Autopsy revealed a remnant tumor in the bile duct (Fig. 7), but no noticeable nodular metastasis was observed, except for a single small metastasis in the lower lobe of his left lung.

## Discussion

We reported an unusual case of biliary dissemination of CRC that caused recurrent intrabiliary growth after hepatectomy. Unfortunately, 3 months postoperatively, he was readmitted for subacute cholangitis and obstructive jaundice due to intrabiliary tumor growth in the remnant liver. Eventually he died of septic liver failure caused by cholangitis. An autopsy revealed a remnant tumor in the bile duct, but no noticeable nodular metastasis was observed. Intrabiliary metastasis of CRC is not typically reported in both Eastern and Western countries [9]. Owing to the rarity of the disease, a majority of published literature comprises either case reports or studies of small case series [5, 7–9, 11, 12]. Reportedly, the prevalence of intrabiliary metastasis by CRC ranges from 3.6–10.6% [9]; however, the prognosis of this disease remains unclear. In Japan, it might be less aggressive compared with usual nodular liver metastasis, and active resection is considered to improve patients’ survival [2, 3, 6]. We decided to perform extended left hepatectomy in this case of biliary dissemination of CRC. In contrast, five cases with poor prognosis have been reported in Korea [11]; these patients did not undergo surgery but palliative procedures, such as PTCD or endoscopic retrograde biliary drainage (ERBD), with a risk of repeated cholangitis. In our case, refractory cholangitis due to intrabiliary tumor growth in the remnant liver caused liver failure similarly. Interestingly, despite the remnant tumor in the bile duct, no life-threatening distant metastasis occurred until our patient’s death. We anticipate that bile duct metastasis is, basically, a sign of less aggressiveness of the tumor; thus, more caution is required to avoid bile duct dissemination at the time of surgery. As this metastatic disease also mimics cholangiocarcinoma, we were unable to differentiate it before resection. Perhaps, the definitive diagnosis of metastatic colonic carcinoma could be attributed to immunohistochemistry, which revealed CDX2^+^, CK20^+^, and CK7^−^ [13, 14]. Fortunately, we could resect the primary lesion in a two-stage operation; however, the management of synchronous liver metastases from CRC remained complex and multidisciplinary [1]. A reliable report from the USA reported the highest number of cases and demonstrated that all prevalence estimates for intrabiliary growth were considerably affected by different clinical management styles for metastatic CRC within each institution [9]. However, the effect of chemotherapy and radiotherapy remains unknown. As mentioned in this case, radiotherapy could have resulted in the tumor shrinkage effect, but it could have been temporary. Perhaps, the complete resection of the intrabiliary growth would have improved the prognosis of our patient.

## Conclusions

Occasionally, the intrabiliary growth of metastatic CRC mimics cholangiocarcinoma and might cause refractory cholangitis. To date, as the effect of chemotherapy or radiotherapy remains uncertain, the complete resection of bile duct tumors is the only method to prevent postoperative cholangitis and could result in a better prognosis.

## Abbreviations

ALP: Alkaline phosphatase
CK: Cytokeratin
CRC: Colorectal cancer
CT: Computed tomography
PTCD: Percutaneous transhepatic cholangio-drainage
